# Interspecific competition affects spore germination and gametophore development of mosses

**DOI:** 10.12688/openreseurope.16004.2

**Published:** 2024-01-22

**Authors:** Jingmin Cheng, Isidora Lončarević, Nils Cronberg

**Affiliations:** 1 School of Environment, Tsinghua University, Beijing, China; 2 Department of Biology, Lund University, Lund, Sweden

**Keywords:** mosses, spore germination, gametophores, competition, allelopathy, Atrichum undulatum, Bryum argenteum, Ceratodon purpureus, Funaria hygrometrica, Hypnum cupressiforme, Leptobryum pyriforme

## Abstract

**Background:**

Interactions between moss species in their earliest growth stages have received little attention. To what extent interspecific competition or priority effects influence spore germination, protonemal development and gametophore emergence is unknown. We evaluated such effects in pairwise interaction between six common bryophyte species: *Atrichum undulatum*, *Bryum argenteum*, *Ceratodon purpureus, Funaria hygrometrica*, *Hypnum cupressiforme*, *Leptobryum pyriforme*

**Methods:**

Interspecific interactions were assessed *in vitro*. Spores were sterilized and sown on agar plates in three treatments: 1) as single species cultures (controls), 2) as pairwise species cultures inoculated simultaneously, and 3) with a time lag of 20 days between species. Data on time needed for spore germination, germination rate, the time needed for gametophore differentiation, number of gametophores per germinated spore and average diameter of colonies were collected. We also performed spore germination tests in single-species cultures at the start and end of the study, as well as tests for density-dependency at spore germination and gametophore formation.

**Results:**

We observed strong pairwise interactive effects when sowing spores of different species simultaneously or with a delay of 20 days. The results indicate that spore germination is often inhibited by interspecific competition. The first species has an advantage as compared to the later colonizing species, i.e., an apparent priority effect. Interspecific interactions were also evident during gametophore development and included both inhibition and facilitation.

**Conclusion:**

We found pronounced differences in the relative performance of species in interaction with other species during spore germination and gametophore formation. Allelopathic effects are the most probable explanation for these observations. Our results under sterile lab conditions are likely to reflect processes that occur in the wild, governing biotic filtering and bryophyte community assembly during primary and secondary colonization.

## Introduction

Plant community assembly – mechanisms by which the local communities are formed from the species pool – have been intensively studied during past decades ( Kraft & Ackerly, 2014). Much emphasis is given to the relative contribution of deterministic and stochastic processes in the search for principles to explain the assembly of local plant communities. Götzenberger *et al.* (2012) conclude from a meta-analysis of 59 articles that “non-random occurrence of plant species is not a widespread phenomenon”, i.e., plant communities often show an individualistic nature.

Interspecific interaction or competition is an important factor determining the structure and dynamics of plant communities ( Aerts, 1999). Due to considerable differences in biology, it has been proposed that competition plays different roles in the community assembly of vascular plants and bryophytes. There are few studies of competition among mosses ( During & van Tooren, 1990), and most are focused on *Sphagnum* and other peat-forming mosses (e.g., Robroek *et al.*, 2007; Rydin, 1986; Sonesson *et al.*, 2002). Some results based on co-occurrence suggest less efficient competition and predominant mutualism in bryophytes ( Steel *et al.*, 2004), whereas other evidence maintain that species exclude each other to the same degree as among vascular plants ( Wilson *et al.*, 1995). A recent study suggests that at the fine scale, environmental filtering prevails in stressful habitats, while competitive interactions appear more important in more favourable conditions ( Monteiro *et al.*, 2023). However, co-occurrence is not evidence of ecological interactions ( Blanchet *et al.*, 2020), and there is a need for more studies with an experimental approach.

Besides biotic and abiotic filtering processes, priority effects could contribute to the diversity of communities dependent on the order and timing in which diaspores of different species arrive at a colonization site ( Fukami *et al.*, 2016). However, priority effects are rarely considered in plant community assembly because such effects may be considered of short duration if the community is subject to pronounced successional changes. The study of priority effects also requires repeated screening during the colonization process.

Ancestors to modern bryophytes were amongst the first plants to colonise terrestrial habitats, and many species still act as primary or secondary colonisers. Most bryophytes are dispersed by spores that are small enough (10–20µm) to be efficiently wind-borne ( Sundberg, 2013). This means that many viable spores of different species continuously disperse, although the composition of the downfall varies due to different spore dispersal phenologies among species. Attempts to experimentally sow spores in natural habitats have largely failed for unclear reasons ( Miles & Longton, 1990; Newton & Mishler, 1994). It is also methodologically challenging to monitor germination of microscopic spores in the field. In contrast, it is often easy to germinate spores *in vitro*. Laboratory culturing has shown that spore germination and protonemal development can be affected by many abiotic factors, e.g., water, light intensity, photoperiod, pH, calcium ions, and plant hormones such as IAA (Indole Acetic Acid) ( Cove *et al.*, 2006; Glime, 2013; Glime, 2015). A comparison of the extant bryophyte community with the propagule rain community (trapped on agar plates) in Canadian boreal forest showed that long-distance dispersal prevails over short-distance dispersal and that species’ ability to produce large amounts of spores, as well as their environmental tolerance during establishment, may have considerable filtering effects ( Barbé *et al.*, 2016).

Interactions between moss species in their earliest growth stages have received little attention ( Watson, 1981). For example, it remains unclear if negative interactions are expressed as exploitive (resource) competition or interference (chemical) competition. Plant allelopathy is defined as interference concerning growth due to chemical interactions between plants and other organisms, mediated by the release of plant-produced bioactive or toxic specialized metabolites referred to as allelochemicals ( Latif *et al.*, 2017). Such metabolites could have beneficial (stimulatory) or detrimental (inhibitory) effects on target organisms ( Cheng & Cheng, 2015; Zhang *et al.*, 2021). Based on studies by ( Bopp, 1959) and her own observations, Watson (1981) suggested that differences among species in arrival time and sensitivity to leaked chemicals could affect the outcome of competitive interactions and, thus, community assembly.

The early development of mosses involves crucial steps, which could be targeted by exuded chemicals with allelopathic effect on neighbouring species. Spore germination, formation of filamentous protonema with two distinct stages (chloronema and caulonema), and initiation of gametophores involve complex physiological processes controlled by internal and external signals involving several phytohormones. As a rule, conspecific moss spores germinate simultaneously with no primary dormancy ( Vesty *et al.*, 2016). It is known from studies of *Physcomitrium patens* that abscisic acid (ABA) has a negative effect, whereas certain diterpenoid hormones have a positive effect on germination ( Vesty *et al.*, 2016). IAA stimulates the transition from chloronema to caulonema cells (but in high concentrations inhibits the normal development of buds); ABA could inhibit this transition, whereas cytokinins are known to enhance bud formation (from chloronemata or caulonemata) ( Decker *et al.*, 2006; Glime, 2013; Reski, 1998).

This project investigated pairwise interactions between six common bryophyte species ( *Atrichum undulatum*, *Bryum argenteum*, *Ceratodon purpureus*, *Funaria hygrometrica*, *Hypnum cupressiforme* and *Leptobryum pyriforme*). We sowed spores on agar plates in sterile conditions and compared their germination and growth, aiming to answer the following three questions: 1) Is it possible to observe interspecific interactions between mosses during spore germination and gametophore development? 2) How are spore germination and protonemal growth affected by interspecific interaction? 3) Is the competitive ability of mosses related to the time when they are sown, i.e., is there a priority effect dependent on the first coloniser? To address these questions, we also performed a spore germination test in single-species cultures at the start and end of the study and checked for density-dependency at spore germination and gametophore formation.

## Methods

### Spore germination, protonemal development and gametophore initiation

Moss spores that deposit on a suitable substrate germinate if enough water is available. Spore germination includes a swelling stage involving water uptake and a distension stage when the cell wall rupture and a germ tube forms ( Mogensen, 1978) that divides to initiate the protonema, which is a 2-dimensional algal-like system of branched filaments. The first protonemal stage is called chloronema and typically has hyaline cell walls, with transverse cross-walls and numerous round chloroplasts. The caulonema develops later, with pigmented cell walls, oblique cross-walls, and fewer and smaller plastids. The caulonema is the primary source of gametophore buds. The cauolonema from a single spore can give rise to multiple gametophores. The gametophore is the 3-dimensional and more persistent life stage of mosses which could form either acrocarpous or pleurocarpous shoot systems, which appear as cushions or tufts respectively as spreading carpets or wefts.

### Study species

We included six common species, *Atrichum undulatum, Bryum argenteum, Ceratodon purpureus, Funaria hygrometrica, Hypnum cupressiforme* and *Leptobryum pyriforme,* which frequently produce sporophytes with overlapping spore dispersal period in the early autumn. It is, therefore, likely that the spores encounter when deposited on bare ground, although the gametophores only occasionally grow together in nature and do not represent any specific moss community. At this stage, it was more important for us that they represent different phylogenetic lineages (different moss families) and growth forms ( Table 1).

**Table 1.** Description of the six study species, sampling coordinates, growth form, morphology and ecology.

Species (Family)	Sampling coordinates	Growth Form	Gametophore size	Ecology
*Atrichum undulatum* (Polytrichaceae)	56°5'32.9"N 13°6'19.9"E	Acrocarp	Stems are up to 7 cm high.	Perennial stayer in shaded, well-drained places.
*Bryum argenteum* (Bryaceae)	55°42'46.6"N 13°12'19"E	Acrocarp	Stems are up to 1–2 cm high.	Colonist on disturbed ground.
*Ceratodon purpureus* (Ditrichaceae)	55°42'46.6"N 13°12'19"E	Acrocarp	Stems are up to 3 cm high.	Colonist in exposed habitats, on disturbed ground.
*Funaria hygrometrica* (Funariaceae)	55°42'44.2"N 13°12'22.3"E	Acrocarp	Stems are about 1 cm high.	Fugitive on exposed and disturbed ground, characteristic of old bonfire sites.
*Hypnum cupressiforme* (Hypnaceae)	56°5'32.9"N 13°6'19.9"E	Pleurocarp	Stems are prostrate and form dense mats.	Perennial stayer on hard surfaces and on ground.
*Leptobryum pyriforme* (Meesiaceae)	55°42'44.2"N 13°12'22.3"E	Acrocarp	Stems are up to 1-2 cm high	Colonist in arable fields and disturbed ground.

### Species collection and preparation of spore suspensions

Fully developed specimens with sporophytes and gametophores were collected from Scania, the southernmost province of Sweden, in October (See Table 1 for full information about sampling sites). First, for each species, a ripe unopened capsule was surface sterilized by dipping in 0.5% NaDCC solution for 2 minutes and then thoroughly washed several times in sterile distilled water. After sterilization, each sporophyte was ruptured with a sterile rod in 0.5 ml sterile distilled water in a separate sterile microtube. The spore suspensions were homogenized by a vortex mixer (VWR; “lab dancer”) and the spore concentration in each suspension was then estimated under a microscope by a haemocytometer. All spore suspensions were diluted to a concentration of approximately 300 spores/100μL. Subsequently, the spore suspensions (one for each species, used throughout the experiment) were stored in darkness in a refrigerator until inoculations.

### Growth medium

We used a mineral nutrient medium by Rudolph *et al.* (1988) originally formulated for nutrient-poor *Sphagnum* cultures, with a pH value of 5.6 after sterilization. We applied this medium in a fourfold concentration solidified with 1 % agar. After sterilization in an autoclave, we distributed the nutrient solution into small Petri dishes (diameter=5 cm). Before starting the experiment, we checked that the spores were viable and able to germinate within a short period (5 days) on agar plates.

### Experimental design and data analysis

**Figure 1.**
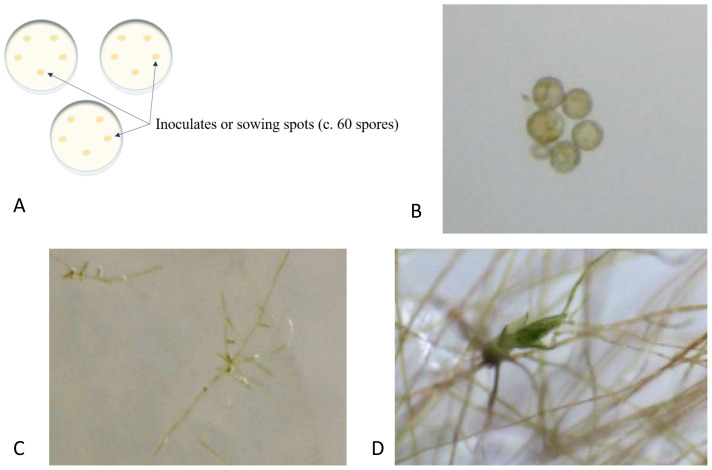
**A**. Illustration of the nested design (for all three treatments) with five separate inoculates on each of the three replicated Petri dishes. **B**– **D**. Moss development exemplified by *Leptobryum pyriforme*. **B**. Mature spores at sowing on agar plate. **C**. Germinated spores forming protonema, seven days after sowing. **D**. Gametophore budding from the protonema three weeks after sowing.

**Figure 2.**
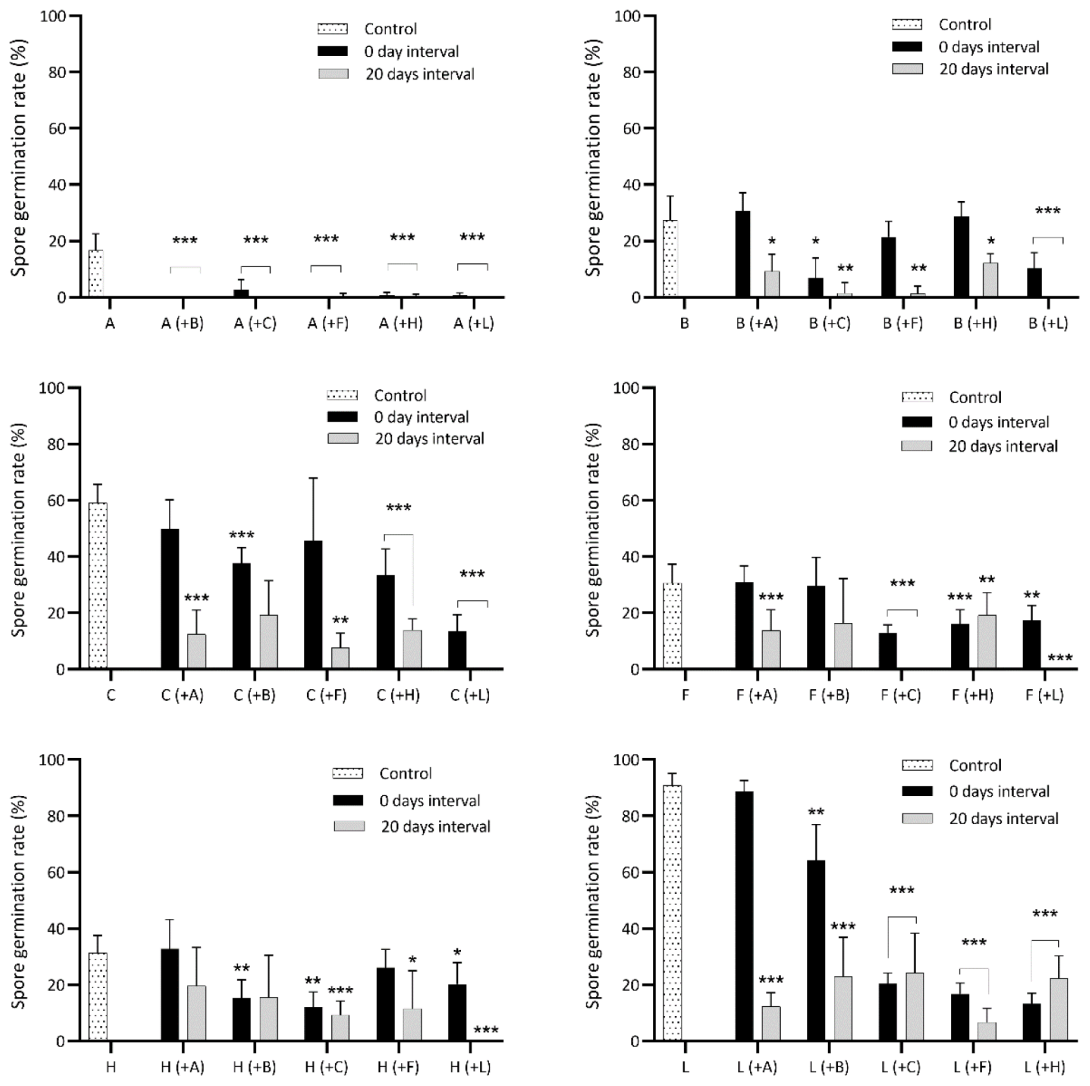
Spore germination rate of six bryophytes when sown in pairwise combinations in axenic culture. Controls are single-species cultures. Each species pair was sown simultaneously and with a delay of 20 days between the first and second species. A, B, C, F, H and L are abbreviations for *A. undulatum, B. argenteum, C. purpureus, F. hygrometrica, H. cupressiforme* and *L. pyriforme*, respectively. Each graph displays the spore germination of controls for a certain species, its spore germination when sown simultaneously with each of the five other species (in parentheses) and its spore germination when sown with a 20-day delay relative to the other species. Bars display mean germination and standard deviation for 15 replicates in a nested design (five replicates within each of three agar plates). The performance of individual species when growing alone (control) versus in combination with another species (sown simultaneously respectively with a 20-day delay) is statistically tested using separate nested ANOVAs. Statistical significance levels p=0.05, p=0.01, p=0.001 are marked “*”, “**”, “***”, respectively. See Table S1– Table S6 for detailed comparison between each species’ control and treatments.

**Figure 3.**
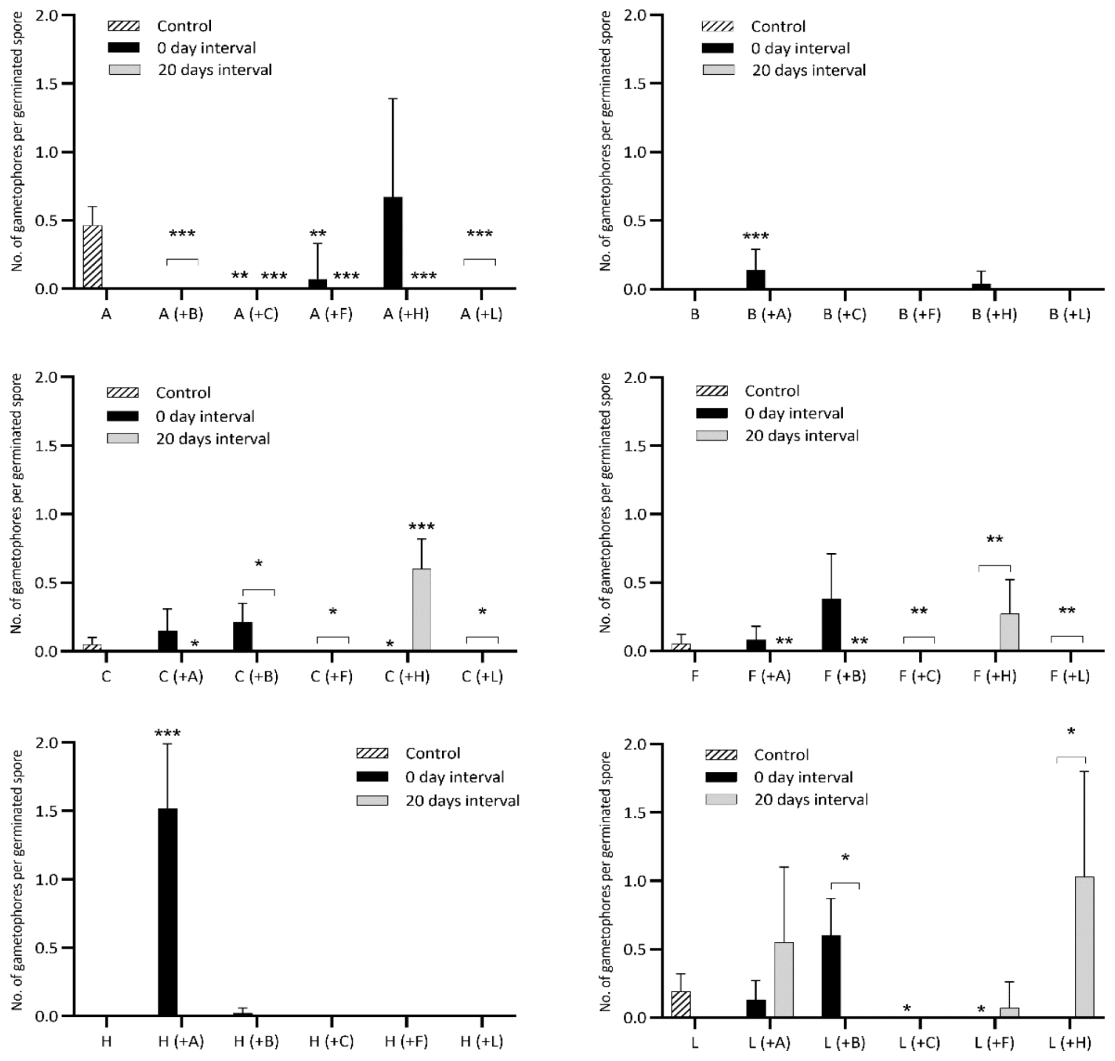
Gametophore budding rate of six bryophytes when sown in pairwise combinations in axenic culture. Controls are single-species cultures. Each species pair was sown simultaneously and with a delay of 20 days between the first and second species. A, B, C, F, H and L are abbreviations for *A. undulatum, B. argenteum, C. purpureus, F. hygrometrica, H. cupressiforme* and *L. pyriforme*, respectively. Each graph displays the gametophore budding in relation to the number of germinated spores of controls for a certain species, its performance when sown simultaneously with each of the five other species (in parentheses) and when sown with a 20-day delay relative to the other species. Bars display mean budding rates and standard deviation for 15 replicates in a nested design (five replicates within each of three agar plates) 40 days after sowing. The performance of individual species when growing alone (control) versus in combination with another species (sown simultaneously respectively with a 20-day delay) is statistically tested using separate nested ANOVAs. Statistical significance levels p=0.05, p=0.01, p=0.001 are marked “*”, “**”, “***”, respectively. See Table S1– S6 for a detailed comparison between each species’ control and treatments.

We sowed spores on agar plates in three treatments: (1) as single species cultures (controls), as pairwise species cultures inoculated (2) simultaneously (spore suspension droplets inoculated on top of each other), and (3) with a time lag of 20 days between the first and second species. The single-species cultures were initiated twice, at the start and end of the experiment, to test for loss of germination capacity during storage. We used a nested design for all three treatments with five separate inoculates on each of the three replicated Petri dishes ( Figure 1A). All inoculates (=sowing spots) consisted of a single 20 µL droplet of spore suspension distributed with a micropipette. Inoculates contained c. 60 spores (since we calibrated the concentration of spore suspension for each species to about 300 spores per 100µL). Inoculates were distributed in a circle inside Petri dishes to maximize the distance between individual inoculates. All Petri dishes with spores were sealed with medical adhesive tape (“Micropore”) since this tape allows for CO ^2^ and O ^2^ diffusion between the Petri dishes and the ambient air. The Petri dishes were cultured in a climate chamber at 14°C, at a diurnal cycle of 16 h light/8 h dark. The Petri dishes were repeatedly randomly redistributed during cultivation to reduce position effects. A light fixture from Topanga provided light with a light emitting plasma (LEP) bulb, which gives a spectrum similar to sunlight at a fairly low light intensity, 50 µmol/m ^2^/s. Spore germination and gametophore budding ( Figure 1 B–D) was scored with a dissecting microscope. For the fast-growing species *B. argenteum*, *C. purpureus*, *F. hygrometrica* and *L. pyriforme*, the frequency of germinated and ungerminated spores was scored on the 4th day after sowing, while for the slower germinating species *A. undulatum* and *H. cupressiforme*, this data was recorded on the 9th day after sowing ( Table 2). The number of gametophore buds for each species was scored on the 40th day after sowing. The exact number of spores in inoculates was somewhat variable. To check if this could be a source of error, we tested for density-dependent spore germination in the control treatment by calculating the correlation between spores sown out for each species in relation to the number of spores germinated. We also tested for density-dependent gametophore budding in the control treatment by correlating the number of gametophore buds to the number of spores germinated. The spore germination rates of individual species were compared between the single-species cultivation condition (controls) and each of the two pairwise cultivation conditions (i.e., simultaneous and 20-day time-lag treatments) separately ( Figure 2, Table S1– Table S6), as well as gametophore budding rates ( Figure 3, Table S1– Table S6), by nested ANOVAs ( McDonald, 2014) using a spreadsheet template, fetched from http://www.biostathandbook.com/nestedanova.html. All statistical tests were pairwise, so the only options were either no significance or significance (positive or negative, dependent if facilitation or suppression occurred) in comparison between control and treatment for each species. The data was normally distributed, and no transformations were needed. In many cases, the difference between the treatment and the control was quite strong without or almost with no germination in the treatments.

**Table 2.** General description of spore size, spore germination and gametophore development for six studied species in single species cultures, mean±standard deviation of 15 replicates in a nested design (five replicates within each of three agar plates).

Species	Spore size (µm)	Germination time (days)	Spore germination rate (%)	Gametophore differentiation time (days)	No. of gametophores per germin-ated spore	Diameter of colony on 15th day (cm)	Diameter of colony on 50th day (cm)	Spore germination rate at the end of experiment (%)
*A. undulatum*	16-21	3-8	16.6 ±5.9	20-28	0.46 ±0.14	N/A	1.2 ±0.03	1.38 ±0.02
*B. argenteum*	10-15	2-4	27.3 ±8.7	N/A	0	0.35 ±0.02	0.87 ±0.02	30.1 ±6.5
*C. purpureus*	10-17	2-4	59.1 ±6.6	20-28	0.05 ±0.05	0.44 ±0.01	0.83 ±0.01	70.8 ±2.4
*F. hygrometrica*	16-24	2-4	30.6 ±6.7	20-28	0.05 ±0.07	0.45 ±0.01	0.67 ±0.00	26.0 ±7.6
*H. cupressiforme*	15-25	3-8	31.4 ±6.2	N/A	0	N/A	0.42 ±0.00	32.0 ±7.9
*L. pyriforme*	9-13	2-4	90.9 ±4.2	20-28	0.19 ±0.13	0.63 ±0.01	1.38 ±0.02	94.4 ±6.29

## Results

For the single-species cultures (controls), data on time needed for spore germination, germination rate, the time needed for gametophore differentiation, gametophore per germinated spore, and average colony diameter on the 15 ^th^ and 50 ^th^ day after sowing are listed in Table 2. Among the six species, *B. argenteum, C. purpureus, F. hygrometrica,* and *L. pyriforme* germinated quickly after sowing, whereas *A. undulatum* and *H. cupressiforme* germinated much slower. The germination rate ranged from c. 90% in *L. pyriforme* to c. 17% in *A. undulatum*.

Some small, brownish spores (lacking chlorophyll) occurring in the samples of *A. undulatum*, *F. hygrometrica* and *H. cupressiforme* were seemingly unable to germinate. Therefore, they were considered unviable and left out when counting the total number of spores.

The rate of colony expansion differed between species. On the 15 ^th^ day after sowing, the protonemata of *A. undulatum* and *H. cupressiforme* did not merge into visible colonies because of slow growth rate after germination, but the other four species formed visible round colonies that could be measured by a ruler ( Table 2), *L. pyriforme* forming the largest. However, on the 50 ^th^ day after sowing, the colonies of *A. undulatum* were distinct, being the second largest among the six species *.*

Most species formed gametophores 20–28 days after sowing; the exceptions were *B. argenteum* and *H. cupressiforme*, which did not develop gametophores at all in single species cultures. *A. undulatum* had the highest amounts of gametophores per germinated spore. The germination rates were unaffected by storage of spore suspensions in a refrigerator for all species except *A. undulatum*, which completely lost germination capacity between the first and the second germination test in single species condition ( Table 2). Conspecific spore germination was generally density-independent at the spore concentrations used in the controls, except for *A. undulatum*, which displayed negative density-dependence, suggesting that the spores first to germinate could suppress the germination of other spores ( Table 3). Similarly, *A. undulatum* was the only species with density-dependent gametophore budding, suggesting that a higher density of germinated spores induced more buds.

**Table 3.** Correlations between total number of spores and germination frequency respectively between number of germinated spores and number of gametophores for single-species cultures. The correlations are based on 15 replicates in a nested design (five replicates within each of three agar plates).

Species	Mean no. of spores	Correlation coefficient	p	Mean no of gametophores	Correlation coefficient	p
*A. undulatum*	77	-0.549	**0.034**	5.7	0,518	**0,048**
*B. argenteum*	51	-0.346	0.201	0	N/A	N/A
*C. purpureus*	49	0.231	0.408	1.4	0.104	0.712
*F. hygrometrica*	57	-0.059	0.835	0.86	-0.204	0.465
*H. cupressiforme*	62	0.132	0.639	0	N/A	N/A
*L. pyriforme*	62	0.021	0.939	9.9	-0.415	0.123

### *Atrichum undulatum* 

When cultured with *B. argenteum* and *F. hygrometrica*, respectively, *A. undulatum* did not germinate, whether the spores were sown simultaneously or with a delay. When sown at the same time as *C. purpureus*, *H. cupressiforme* and *L. pyriforme*, the germination rate of *A. undulatum* decreased significantly. When sown 20 days later than the three species above, *A. undulatum* showed no germination at all. Considering that *A. undulatum* was the only species that lost spore germinability during storage, the data from the 20 days delay treatment cannot be trusted ( Figure 2; Table S1). Only when sown simultaneously with *H. cupressiforme* did *A. undulatum* form gametophores in similar frequency as the control group, and in almost all other cases, no gametophores formed at all ( Figure 3; Table S1).

### *Bryum argenteum* 

When cultured at the same time as all other five species *, B. argenteum* germinated comparatively well, but its germination rate decreased significantly when grown with *C. purpureus* and *L. pyriforme*. When sown 20 days later than other species, the germination rate of *B. argenteum* decreased strongly in all species treatments; combined with *F. hygrometrica* and *L. pyriforme* it failed completely to germinate ( Figure 2, Table S2). In controls, *B. argenteum* did not form gametophores within 40 days, but when sown at the same time as *A. undulatum,* it did develop gametophores within this period. No gametophores were formed in any cultures with 20 days delay ( Figure 3, Table S2).

### *Ceratodon purpureus* 

**Figure 4.**
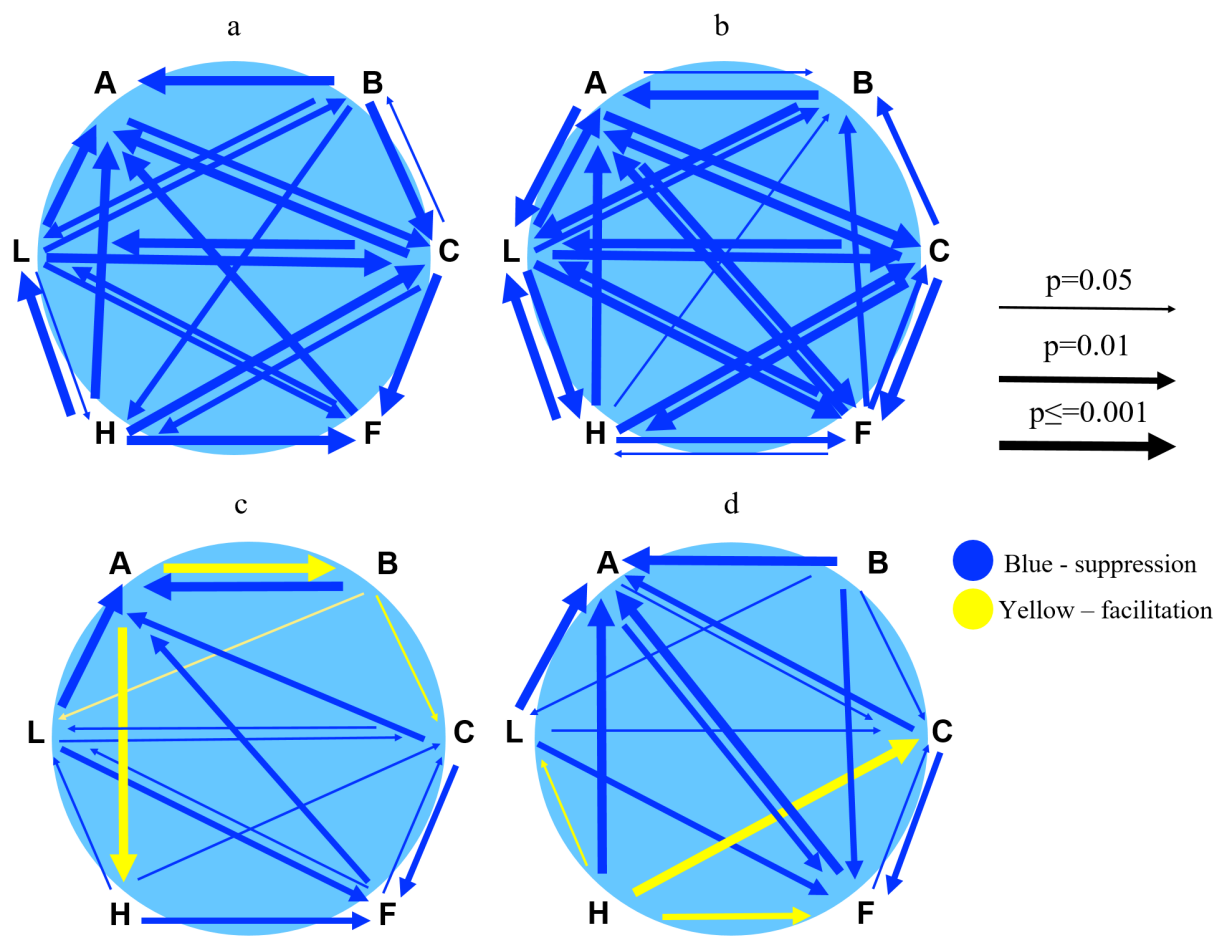
Pairwise interactions between species. **a**. Matrix displaying interaction in terms of spore germination rate between six species when sown pairwise and simultaneously. **b**. Matrix displaying interaction in terms of spore germination rate between six species when sown pairwise and one species in the pair 20 days later than the first. **c**. Matrix displaying interaction in terms of gametophore budding relative to the number of germinated spores between six species when sown pairwise and simultaneously. **d**. Matrix displaying interaction in terms of gametophore budding relative to number of germinated spores between six species when sown pairwise and one species in the pair with 20 days delay. A, B, C, F, H and L are abbreviations for *A. undulatum, B. argenteum, C. purpureus, F. hygrometrica, H. cupressiforme* and *L. pyriforme*, respectively. Thickness of arrows is proportional to the degree of significance (p=0.05, thin; p=0.01, intermediate; p=0.001, thick). The arrowhead is pointing to the species that is affected by the species at the tail of the arrow. Blue colour indicates suppression and yellow colour indicates facilitation. Arrows are not displayed for non-significant interactions, i.e., when there was no difference between the test and the control.

When sown at the same time as the other five species *,* the germination rate of *C. purpureus* decreased significantly. When sown 20 days later than the others, its germination rate became even lower *,* especially with *L. pyriforme*, *C. purpureus* did not germinate at all ( Figure 2, Table S3). Only when cultured at the same time as *B. argenteum* and 20 days later than *H. cupressiforme* did *C. purpureus* form gametophores, but then with a number of gametophores per germinated spore that was significantly higher than in the controls, and strongly so with *H. cupressiforme*. In all other combinations *C. purpureus* did not form gametophores at all ( Figure 3, Table S3). Thus, the development of gametophores of *C. purpureus* could either be promoted or inhibited in co-occurrence with other species ( Figure 4).

### *Funaria hygrometrica* 

When sown at the same time as the other species the germination rate of *F. hygrometrica* decreased significantly. When sown 20 days later than the other species, germination of *F. hygrometrica* in all treatments was strongly inhibited, with no germination at all when cultured with *C. purpureus* and *L. pyriforme* ( Figure 2, Table S4). Only when sown 20 days later than *H. cupressiforme* did *F. hygrometrica* form gametophores and at a significantly higher rate than in the control ( Figure 3 and Figure 4, Table S4).

### *Hypnum cupressiforme* 

When sown at the same time as other species *,* the germination rate of *H. cupressiforme* decreased significantly. When sown 20 days later than other species, *H. cupressiforme* was strongly inhibited in the spore germination stage, especially with *L. pyriforme,* it did not germinate at all ( Figure 2, Table S5). *H. cupressiforme* formed gametophores only in treatment where it was sown simultaneously with *A. undulatum,* suggesting a promoting effect of *A. undulatum* on its gametophore development, ( Figure 3 and Figure 4, Table S5).

### *Leptobryum pyriforme* 

In controls, *L. pyriforme* showed a germination rate of 95%, and this germinability was retained nearly so with the fast germinator *B. argenteum*, whereas germinability dropped significantly to around 10-20% with the remaining species. When sown 20 days later than the other five species, the germination rate of *L. pyriforme* was generally around (5-)10-20% ( Figure 2, Table S6). When cultured with *C. purpureus*, no matter if sown at the same time or 20 days later, *L. pyriforme* did not form gametophores at all, and almost so with *F. hygrometrica*. When cultured simultaneously with *H. cupressiforme*, no gametophores were formed, but when cultured with 20-day delay, a significantly higher frequency of gametophores budded than the controls ( Figure 3). Conversely, gametophores emerged more frequently than the controls when spores were sown at the same time as *B. argenteum* but were absent when sown with a delay ( Figure 3, Table S6).

We have summarized the pairwise interactions between species in Figure 4 (a–d). The interactions display a complex pattern involving suppression (blue arrows) and facilitation (yellow arrows).

## Discussion

Recent studies of bryophyte community assembly based on occurrence data stress the importance of habitat filtering as well as dispersal capacity ( Barbé *et al.*, 2016; Lönnell & Hylander, 2018; Tiselius *et al.*, 2019). Whereas the abiotic requirements are relatively well characterized for many species (e.g., for pH values; Tyler & Olsson, 2016), the biotic filtering processes following spore deposition are poorly known. When sown simultaneously, we show pronounced biotic interactions at spore germination involving inhibition of one or the other species. Such effects would impose filtering effects if occurring under natural conditions ( Figures 4a, c). The effects were even more evident when we sowed spores with 20 days delay, indicating apparent priority effects, e.g., the dominance of the species first to colonise ( Figures 4b, d). This is in line with observations of Watson (1981) suggesting that six Polytrichum species differ in their abilities to exclude each other during the juvenile phase. We also observe that some mosses can accelerate the speed of the formation of gametophores in interaction with another species (facilitation) ( Figures 4c and 4d).

### Density-dependence does not influence the results

Negative density-dependent effects occur commonly in vascular plants when crowded seedlings compete for resources and it seems that the stronger this mechanism is, the higher the plant diversity becomes at the global scale ( LaManna *et al.*, 2017). Negative density-dependent effects are likely to occur in the early colonization of bryophytes. For example, it is known that high spore densities may cause the failure of caulonema differentiation (self-inhibition) ( Zamfir & Goldberg, 2000), which might prevent intraspecific competition among protonemata. Also, Bopp (1959) and later Watson (1981) found that single spores of Funaria hygrometrica sown at some distance formed separate protonema that kept apart when encountering, whereas spores sown together freely merged to form a common protonema. We did not see density-dependency in our study at the applied densities, except for *A. undulatum*, which displayed negative density-dependence for spore germination and positive density-dependence at gametophore budding ( Table 3), so this factor is unlikely to affect the results.

### Spore germination time and lateral expansion differ among species

It is known from the literature (summarized by Glime, 2015) that germination time varies with size, age, available light, water, and nutrients. Acrocarpous species benefit from growing in dense cushions and a single protonema can produce multiple gametophytic buds. In the single species controls, we found that *B. argenteum*, *C. purpureus*, *F. hygrometrica* and *L. pyriforme* germinated as fast as three days after sowing, whereas *A. undulatum* and *H. cupressiforme* needed about seven days ( Table 2). Rapid germination would mean that a species could quickly occupy the space (exploitive competition) and earlier turn on the production of allelochemicals (inducing interference competition). In our study, the slowly germinating *H. cupressiforme* performed well when sown simultaneously with the other species ( Figure 2) but largely failed to produce gametophore buds ( Figure 3). In contrast, the other slow germinator, *A. undulatum* displayed reduced germination, or none at all, when sown simultaneously with the other species ( Figure 2). We could see that species showed a different capacity for lateral expansion in single-species cultures, *L. pyriforme* and *A. undulatum* having the most vigorous expansion after 50 days ( Table 2).

Except for *A. undulatum*, the germination rate of spores from other species did not decrease after two months storage in 4°C in darkness. The spore germination rate was relatively low for the studied species (except *L. pyriforme*) in single-species cultures, maybe due to dormancy induced by storage, but this is unlikely to affect the results since the germination rate remained stable over time (except for *A. undulatum*). Data on the longevity of moss spores seems scarce ( Pence, 2004) and most studies have tested survival in dry conditions ( Proctor *et al.*, 2007), so it is an open question how long they remain viable under cool and dark conditions and to what extent it differs within species.

### Exploitive competition in bryophytes

Grime (1973) defined competition as “the tendency of neighbouring plants to utilize the same quanta of light, ion of a mineral nutrient, molecule of water, or volume of space”. He also classified plants into three groups with distinct strategies in interspecific interaction: competitive, stress tolerant and ruderal ( Grime, 1977). Bryophytes were previously considered ruderal or stress-tolerant due to their small size and low growth rate; however, competition has been shown to be important in structuring bryophyte communities similar to vascular plant communities ( Ma *et al.*, 2020; Mulligan & Gignac, 2002; Rydin, 1997). In our study, *A. undulatum* had the lowest spore germination rate ( Table 2, Figure 2) and the shortest spore longevity. It also germinated comparatively slowly and responded negatively to crowding. Taken together, it appears that *A. undulatum* is a poor competitor during establishment, as Miles and Longton (1987) concluded from germination studies under field conditions.

Inhibition of spore germination could result from competition for resources such as water, nutrients, and light (exploitive competition). In our experiments, we expect low competition for water and light, the agar medium consisting of water to 98% and being essentially translucent. Competition for nutrients is more realistic since our culture medium is rather low in minerals. We found that *L. pyriforme*, *C. purpureus*, and *F. hygrometrica* occupied the space quicker than *A. undulatum*, *B. argenteum*, and *H. cupressiforme*, suggesting a stronger competitive ability during early colonization.

### The case for interference competition

Although most bryophyte studies concern exploitive competition, there is growing evidence that interference competition, in the form of chemical interference (allelopathy), occurs as well ( Liu *et al.*, 2020a). For example, growing protonema secretes morphogenetic substances, which regulate the development of other filaments and coordinate the growth and differentiation of neighbouring plants ( Reski, 1998). Recent studies of peat mosses have revealed plant-plant interspecific interaction mediated through volatile organic compounds (VOCs) ( Vicherová *et al.*, 2020) and also that leachates can affect the microbiome, possibly influencing plant fitness and interspecific competition ( Hamard *et al.*, 2019).

It is generally experimentally challenging to distinguish between exploitive and interference competition in natural plant communities ( He *et al.*, 2012). Both can operate simultaneously and interact as shown in studies of the grass *Lolium rigidum* Gaud. and the soy bean *Glycine max* L. ( San Emeterio *et al.*, 2007). In peatland ecosystems, two closely related co-occurring *Sphagnum* species (from hollow and hummock habitats) showed niche differentiation along a water table gradient due to both resource competition and allelopathic effects ( Liu *et al.*, 2020b).

Some observations strongly suggest that chemical (allelopathic) interactions play a vital role in our study. First, the responses were triggered at early spore germination before any resource limitations could arise. Second, facilitation occurred in some pairwise combinations, an unlikely outcome of resource scarcity. As displayed in our matrix figures ( Figure 4a and 4b), most interactions during spore germination involve inhibitory effects. Several compounds with negative allelopathic activity (inhibiting germination, growth, and establishment of surrounding plants) have been discovered in bryophytes ( Basile *et al.*, 2003; Kato-Noguchi & Seki, 2010; Kato-Noguchi *et al.*, 2010; Nozaki *et al.*, 2007).

On the other hand, we also observed clear beneficial effects (facilitation), particularly during gametophore formation. Notably, *A. undulatum* and *H. cupressiforme* were involved in such interactions ( Figure 4). Both *B. argenteum* and *H. cupressiforme* failed to form gametophores in controls. However, they succeeded in doing so when cultured simultaneously with *A. undulatum*, indicating that formation of gametophores was enhanced or speeded up ( Figure 3). Rapid formation of gametophores may help mosses occupy new habitats because of earlier resistance to drought and enhanced performance in competition for light.

The roles of competition and facilitation have been assessed in communities of both vascular plants and bryophytes ( Bu *et al.*, 2013; Mulder *et al.*, 2001; Pedersen *et al.*, 2001; Rixen & Mulder, 2005), ideally together with environmental factors ( Callaway & Walker, 1997). Interspecific facilitation in bryophytes is mainly connected to abiotic factors (light, water levels, different seasons), while intraspecific facilitation is influenced by density-dependent effects and environmental variation ( Cornelissen *et al.*, 2007; Pedersen *et al.*, 2001). According to the stress-gradient hypothesis ( Bertness & Callaway, 1994), the relative importance of competition decreases, and facilitation increases with an increase in abiotic stress. However, the opposite effect was observed in a *Sphagnum* study ( Bu *et al.*, 2013). There are some reports of facilitative effects of bryophytes on vascular plants (e.g., Lett *et al.*, 2018; Soudzilovskaia *et al.*, 2011). Reports that support chemical facilitation in bryophyte communities are few; we have only found two such studies, but they reported contradictory results between laboratory and field experiments and, thus, dependency on environmental conditions ( Liu *et al.*, 2020a; Liu *et al.*, 2020b) which suggests a need for further investigation.

## Concluding remarks

Bryophytes (mosses, liverworts, and hornworts) are likely to have been competing for space ever since the groups originated and diversified during the Silurian and Devonian periods, long before the advent of seed plants. Our study gives a glimpse of interactions during spore germination and gametophore formation, which are likely to impose profound filtering effects when mosses establish in a new habitat, not the least in places far away from the parent colony ( Odu, 1979). Our results also align with the discrepancy Barbé *et al.* (2016) found between extant bryophyte communities relative to the ones they observed to emerge from aerially dispersed propagules trapped on agar plates. Their data suggest that the spore rain from distant sources often has a stronger influence on community formation than nearby propagule dispersal due to largely unknown filtering mechanisms. 

The diversity of compounds produced inside bryophyte tissues is extensively investigated (see, e.g., Asakawa *et al.*, 2013). In contrast, substances released by bryophytes to the ambient environment are almost completely neglected. We envision that bryophyte exudation might compare to rhizosphere exudation in vascular plants, a research area that has exploded in recent years ( Bais *et al.*, 2006; Wang *et al.*, 2021).

This study is restricted in the sense that we made it under sterile conditions, with a narrow span of microclimatic variation, which means that most natural stressors and biotic interactions are excluded. Although it is conventional to study interactions between species by comparing their performance in two-species communities with that in single-species culture ( Jolliffe, 2000), we acknowledge that this gives a simplified picture of community assembly. We observe considerable differentiation in interactions between species, but the data set is too limited to draw any conclusions related to phylogenetic lineages, habitats or life forms. We avoided focusing on any particular moss community because naturally co-occurring species could have evolved specific adaptations. Oour results exemplify more generalized responses unrelated to any specific habitat context. The species included have relatively small spores but species belonging to the life strategy categories of shuttle species (annual, short-lived and perennial shuttles) *sensu* During (1979) generally have larger spores as an adaption to frequent disturbance and local secondary recolonization. In future studies, it would be interesting to test if their larger spores infer advantages in exploitive and interference competition. As our study had a narrow genetic basis, including spores from a single sporophyte for each species, testing for variation within species is also desirable.

Our study paves the way for novel experimental designs, which could be used to identify potential bryophyte-released allelochemicals in combination with advanced analytical chemistry techniques. For example, it would be possible to test if the composition of exuded substances differs when the species grow alone or in combination with other species. Such studies, including the comparison of effects under field and laboratory conditions, are crucial to getting deeper insights into the role of interactions and allelopathy in the assembly of bryophyte communities.

## Figures and Tables

**Table S1.** Spore germination rate (no. of germinated spores/total no. spores) and gametophore production (no. of gametophores/no. of germinated spores) for *Artrichum undulatum* (A) as cultured in pairwise combinations with five other species (B = *Bryum argenteum*; C = *Ceratodon purpureus*; F = *Funaria hygrometrica*; H = *Hypnum cupressiforme*; L = *Leptobryum pyriforme*) sown simultaneously (0 days) or with a delay of 20 days (with *A. undulatum* as the second species). Mean value ± standard deviation (SD) is indicated for spore germination rate and gametophore production. The cultures were carried out in a nested design (five replicates within each of three agar plates). The scores from pairwise cultures were analysed against single species cultures using a nested ANOVA. Statistical significance levels p=0.05, p=0.01, p=0.001 are marked “*”, “**”, “***”, respectively. S or F in the table stands for the suppression or facilitation in case of significant treatment.

Dependent	Species Treatment	Time Interval	Average±SD	df	F-value	p-value
**Spore germination rate**	A(with B)	0 day S	0	29	147.9	2.6 10 ^-4^ ***
		20 days S	0	29	147.9	2.6 10 ^-4^ ***
	A(with C)	0 day S	2.83%±3.43%	29	84.55	7.8 10 ^-4^ ***
		20 days S	0	29	147.9	2.6 10 ^-4^ ***
	A(with F)	0 day S	0.14%±0.38	29	145.0	2.7 10 ^-4^ ***
		20 days S	0	29	147.9	2.6 10 ^-4^ ***
	A(with H)	0 day S	0.84%±0.83%	29	127.0	3.5 10 ^-4^ ***
		20 days S	0	29	147.9	2.6 10 ^-4^ ***
	A(with L)	0 day S	0.49%±0.78%	29	134.2	3.2 10 ^-4^ ***
		20 days S	0	29	147.9	2.6 10 ^-4^ ***
**No. of gametophores** **per germinated spore**	A(with B)	0 day S	0	29	231.6	1.1 10 ^-4^ ***
		20 days S	0	29	231.6	1.1 10 ^-4^ ***
	A(with C)	0 day S	0	29	30.8	0.0052 **
		20 days S	0	29	231.6	1.1 10 ^-4^ ***
	A(with F)	0 day S	0.07±0.26	29	30.8	0.0052 **
		20 days S	0	29	231.6	1.1 10 ^-4^ ***
	A(with H)	0 day	0.67±0.72	29	1.954	0.235 ns
		20 days S	0	29	231.6	1.1 10 ^-4^ ***
	A(with L)	0 day S	0	29	231.6	1.1 10 ^-4^ ***
		20 days S	0	29	231.6	1.1 10 ^-4^ ***

**Table S2.** Spore germination rate (no. of germinated spores/total no. spores) and gametophore production (no. of gametophores/no. of germinated spores) for *Bryum argenteum* (B) as cultured in pairwise combinations with five other species (A = *Atrichum undulatum*; C = *Ceratodon purpureus*; F = *Funaria hygrometrica*; H = *Hypnum cupressiforme*; L = *Leptobryum pyriforme*) sown simultaneously (0 days) or with a delay of 20 days (with *B. argenteum* as the second species). Mean value ± standard deviation (SD) is indicated for spore germination rate and gametophore production. The cultures were carried out in a nested design (five replicates within each of three agar plates. The scores from pairwise cultures were analysed against single species cultures using a nested ANOVA. Statistical significance levels p=0.05, p=0.01, p=0.001 are marked “*”, “**”, “***”, respectively. S or F in the table stands for suppression or facilitation in case of significant treatment.

Dependent	Species Treatment	Time Interval	Average±SD	df	F-value	p-value
**Spore germination rate**	B(with A)	0 day	30.65%±6.45%	29	0.5664	0.493 ns
		20 days S	9.24%±6.07%	29	15.99	0.016 *
	B(with C)	0 day S	6.81%±7.15%	29	14.00	0.020 *
		20 days S	1.49%±3.73%	29	33.48	0.0044 **
	B(with F)	0 day	21.43%±5.54%	29	1.707	0.261 ns
		20 days S	1.28%±2.65%	29	32.49	0.0047 **
	B(with H)	0 day	28.73%±5.15%	29	0.1061	0.761 ns
		20 days S	12.15%±3.34%	29	10.77	0.0304 *
	B(with L)	0 day S	10.29%±5.47%	29	12.80	0.00232 **
		20 days S	0	29	38.79	0.0035 **
**No. of gametophores** **per germinated spore**	B(with A)	0 day F	0.14±0.15	29	163.4	2.2 10 ^-4^ ***
		20 days	0	29	N/A	N/A
	B(with C)	0 day	0	29	N/A	N/A
		20 days	0	29	N/A	N/A
	B(with F)	0 day	0	29	N/A	N/A
		20 days	0	29	N/A	N/A
	B(with H)	0 day	0.04±0.09	29	2.9174	0.163 ns
		20 days	0	29	N/A	N/A
	B(with L)	0 day	0	29	N/A	N/A
		20 days	0	29	N/A	N/A

**Table S3.** Spore germination rate (no. of germinated spores/total no. spores) and gametophore production (no. of gametophores/no. of germinated spores) for *Ceratodon purpureus* (C) as cultured in pairwise combinations with five other species (A = *Atrichum undulatum*; B = *Bryum argenteum*; F = *Funaria hygrometrica*; H = *Hypnum cupressiforme*; L = *Leptobryum pyriforme*) sown simultaneously (0 days) or with a delay of 20 days (with *C. purpureus* as the second species). Mean value ± standard deviation (SD) is indicated for spore germination rate and gametophore production. The cultures were carried out in a nested design (five replicates within each of three agar plates). The scores from pairwise cultures were analysed against single species cultures using a nested ANOVA. Statistical significance levels p=0.05, p=0.01, p=0.001 are marked “*”, “**”, “***”, respectively. S or F in the table stands for suppression or facilitation in case of significant treatment.

Dependent	Species Treatment	Time Interval	Average±SD	df	F-value	P-value
**Spore germination rate**	C(with A)	0 day	49.89%±10.32%	29	4.800	0.094 ns
		20 days S	12.35%±8.66%	29	128.8	3.4 10 ^-4^ ***
	C(with B)	0 day S	37.71%±5.52%	29	124.4	3.7 10 ^-4^ ***
		20 days	19.33%±12.11%	29	0.9701	0.380
	C(with F)	0 day	45.59%±22.33%	29	1.74	0.258 ns
		20 days S	7.66%±5.14%	29	35.40	0.0040 **
	C(with H)	0 day S	33.47%±9.27%	29	121.6	3.8 10 ^-4^ ***
		20 days S	13.89%±4.00%	29	1169	4.3 10 ^-6^ ***
	C(with L)	0 day S	13.58%±5.83%	29	743.2	1.1 10 ^-5^ ***
		20 days S	0	29	21.51	9.7 10 ^-4^ ***
**No. of gametophores** **per germinated spore**	C(with A)	0 day	0.15±0.16	29	2.004	0.230 ns
		20 days S	0	29	19.71	0.011 *
	C(with B)	0 day F	0.21±0.14	29	15.43	0.017 *
		20 days S	0	29	19.71	0.011 *
	C(with F)	0 day S	0	29	19.71	0.011 *
		20 days S	0	29	19.71	0.011 *
	C(with H)	0 day S	0	29	19.71	0.011 *
		20 days F	0.43±0.18	29	94.94	6.2 10 ^-4^ ***
	C(with L)	0 day S	0	29	19.71	0.011 *
		20 days S	0	29	19.71	0.011 *

**Table S4.** Spore germination rate (no. of germinated spores/total no. spores) and gametophore production (no. of gametophores/no. of germinated spores) for *Funaria hygrometrica* (F) as cultured in pairwise combinations with five other species (A = *Atrichum undulatum*; B = *Bryum argenteum*; C= *Ceratodon purpureus;* H = *Hypnum cupressiforme*; L = *Leptobryum pyriforme*) sown simultaneously (0 days) or with a delay of 20 days (with *F. hygrometrica* as the second species). Mean value ± standard deviation (SD) is indicated for spore germination rate and gametophore production. The cultures were carried out in a nested design (five replicates within each of three agar plates). The scores from pairwise cultures were analysed against single species cultures using a nested ANOVA. Statistical significance levels p=0.05, p=0.01, p=0.001 are marked “*”, “**”, “***”, respectively. S or F in the table stands for the suppression or facilitation in case of significant treatment.

Dependent	Species Treatment	Time Interval	Average±SD	df	F-value	P-value
**Spore germination rate**	F(with A)	0 day	30.91%±5.77%	29	0.0250	0.882 ns
		20 days S	13.78%±7.38%	29	88.95	7.0 10 ^-4^ ***
	F(with B)	0 day	29.60%±10.09%	29	0.0718	0.802 ns
		20 days	16.35%±15.82%	29	6.482	0.0636 ns
	F(with C)	0 day S	12.93%±2.78%	29	117.9	4.1 10 ^-4^ ***
		20 days S	0	29	553.6	1.9 10 ^-5^ ***
	F(with H)	0 day S	16.13%±4.97%	29	99.9	5.6 10 ^-4^ ***
		20 days S	19.21%±7.98%	29	36.94	0.0037 **
	F(with L)	0 day S	17.31%±5.29%	29	70.36	0.0011 **
		20 days S	0	29	553.6	1.9 10 ^-5^ ***
**No. of gametophores** **per germinated spore**	F(with A)	0 day	0.08±0.10	29	0.3168	0.604 ns
		20 days S	0	29	33.06	0.0045 **
	F(with B)	0 day	0.38±0.33	29	4.247	0.108 ns
		20 days S	0	29	33.06	0.0045 **
	F(with C)	0 day S	0	29	33.06	0.0045 **
		20 days S	0	29	26.39	0.0068 **
	F(with H)	0 day S	0	29	33.06	0.0045 **
		20 days F	0.27±0.25	29	11.146	0.002 **
	F(with L)	0 day S	0	29	33.06	0.0045 **
		20 days S	0	29	33.06	0.0045 **

**Table S5.** Spore germination rate (no. of germinated spores/total no. spores) and gametophore production (no. of gametophores/no. of germinated spores) for *Hypnum cupressiforme* (H) as cultured in pairwise combinations with five other species (A = *Atrichum undulatum*; B = *Bryum argenteum*; C= *Ceratodon purpureus;* F = *Funaria hygrometrica*; L = *Leptobryum pyriforme*) sown simultaneously (0 days) or with a delay of 20 days (with *H. cupressiforme* as the second species). Mean value ± standard deviation (SD) is indicated for spore germination rate and gametophore production. The cultures were carried out in a nested design (five replicates within each of three agar plates). The scores from pairwise cultures were analysed against single species cultures using a nested ANOVA. Statistical significance levels p=0.05, p=0.01, p=0.001 are marked “*”, “**”, “***”, respectively. S or F in the table stands for suppression or facilitation in case of significant treatment.

Dependent	Species Treatment	Time Interval	Average±SD	df	F-value	P-value
**Spore germination rate**	H(with A)	0 day	32.87%±10.32%	29	0.0559	0.825 ns
		20 days	26.27%±29.95%	29	0.2009	0.672 ns
	H(with B)	0 day S	15.42%±6.41%	29	26.14	0.0069 **
		20 days	15.63%±14.88%	29	3.549	0.133 ns
	H(with C)	0 day S	12.12%±5.37%	29	53.24	0.0019 **
		20 days S	9.33%±4.89%	29	255.2	9.0 10 ^-5^ ***
	H(with F)	0 day	26.12%±6.51%	29	5.933	0.072 ns
		20 days S	11.58%±13.40%	29	12.23	0.025 *
	H(with L)	0 day S	20.28%±7.65%	29	16.70	0.015 *
		20 days S	0	29	3097	6.2 10 ^-7^ ***
**No. of gametophores** **per germinated spore**	H(with A)	0 day F	1.52±0.47	29	222.6	1.2 10 ^-4^ ***
		20 days	0	29	N/A	N/A
	H(with B)	0 day	0.02±0.04	29	2.496	0.189 ns
		20 days	0	29	N/A	N/A
	H(with C)	0 day	0	29	N/A	N/A
		20 days	0	29	N/A	N/A
	H(with F)	0 day	0	29	N/A	N/A
		20 days	0	29	N/A	N/A
	H(with L)	0 day	0	29	N/A	N/A
		20 days	0	29	N/A	N/A

**Table S6.** Spore germination rate (no. of germinated spores/total no. spores) and gametophore production (no. of gametophores/no. of germinated spores) for *Leptobryum pyriforme* (L) as cultured in pairwise combinations with five other species (A = *Atrichum undulatum*; B = *Bryum argenteum*; C= *Ceratodon purpureus;* F = *Funaria hygrometrica*; H = *Hypnum cupressiforme*) sown simultaneously (0 days) or with a delay of 20 days (with *L. pyriforme* as the second species). Mean value ± standard deviation (SD) is indicated for spore germination rate and gametophore production. The cultures were carried out in a nested design (five replicates within each of three agar plates). The scores from pairwise cultures were analysed against single species cultures using a nested ANOVA. Statistical significance levels p=0.05, p=0.01, p=0.001 are marked “*”, “**”, “***”, respectively. S or F in the table stands for suppression or facilitation in case of significant treatment.

Dependent	Species Treatment	Time Interval	Average ± SD	df	F-value	p-value
**Spore germination rate**	L(with A)	0 day	88.69%±3.91%	29	3.614	0.130 ns
		20 days S	12.40%±4.80%	29	3818	4.1 10 ^-7^ ***
	L(with B)	0 day S	64.25%±12.68%	29	28.43	0.0060 **
		20 days S	22.98%±13.89%	29	182.2	1.7 10 ^-4^ ***
	L(with C)	0 day S	20.39%±3.82%	29	2448	1.0 10 ^-6^ ***
		20 days S	24.22%±14.13%	29	165.8	2.1 10 ^-4^ ***
	L(with F)	0 day S	16.77%±3.82%	29	10034	5.6 10 ^-8^ ***
		20 days S	7.45%±4.77%	29	1593	2.3 10 ^-6^ ***
	L(with H)	0 day S	13.29%±3.77%	29	5100	2.3 10 ^-7^ ***
		20 days S	22.40%±7.86%	29	697.9	1.2 10 ^-5^ ***
**No. of gametophores** **per germinated spore**	L(with A)	0 day	0.13±0.14	29	0.734	0.439 ns
		20 days	0.55±0.55	29	2.468	0.191 ns
	L(with B)	0 day F	0.60±0.27	29	11.60	0.027 *
		20 days	0	29	8.253	0.045 *
	L(with C)	0 day S	0	29	8.253	0.045 *
		20 days	0	29	1.655	0.268 ns
	L(with F)	0 day S	0	29	8.253	0.045 *
		20 days	0.07±0.19	29	4.109	0.052
	L(with H)	0 day S	0	29	8.253	0.045 *
		20 days F	1.03±0.77	29	10.97	0.030 *

## Data Availability

Dryad. “Spore germination and gametophore development of mosses in response to competition”, licensed under a CC0 1.0 Universal (CC0 1.0) Public Domain Dedication license, doi: 10.5061/dryad.7d7wm380t.
