# Supplementary material for: Interspecific competition affects spore germination and gametophore development of mosses
Source: Open Res Eur. 2024 Jan 22;3:91. Originally published 2023 Jun 12. [Version 2] doi: 10.12688/openreseurope.16004.2 (PMC10558986; doi:10.12688/openreseurope.16004.2)
Supplement: Peer review history

# Reviewer report: Reviewer response for version 2

**DOI:** 10.21956/openreseurope.18502.r37650

**Published:** 2024-05-31

**Authors:** Marko S. Sabovljevic

**Affiliations:** 1 Institute of Botany and Botanical Garden Jevremovac, Faculty of Biology, University of Belgrade, Belgrade, Serbia

The MS can be approved.

Is the study design appropriate and does the work have academic merit?

Partly

Is the work clearly and accurately presented and does it cite the current literature?

No

If applicable, is the statistical analysis and its interpretation appropriate?

I cannot comment. A qualified statistician is required.

Are all the source data underlying the results available to ensure full reproducibility?

Partly

Are the conclusions drawn adequately supported by the results?

Partly

Are sufficient details of methods and analysis provided to allow replication by others?

No

Reviewer Expertise:

Bryophyte Biology

I confirm that I have read this submission and believe that I have an appropriate level of expertise to confirm that it is of an acceptable scientific standard.

# Reviewer report: Reviewer response for version 2

**DOI:** 10.21956/openreseurope.18502.r37651

**Published:** 2024-02-26

**Authors:** Jennifer K. Rowntree

**Affiliations:** 1 University of Plymouth, Plymouth, England, UK

I am satisfied with the authors responses to my comments.

Is the study design appropriate and does the work have academic merit?

Yes

Is the work clearly and accurately presented and does it cite the current literature?

Partly

If applicable, is the statistical analysis and its interpretation appropriate?

Partly

Are all the source data underlying the results available to ensure full reproducibility?

Yes

Are the conclusions drawn adequately supported by the results?

Yes

Are sufficient details of methods and analysis provided to allow replication by others?

Partly

Reviewer Expertise:

Ex situ bryophyte conservation and in vitro culture of bryophytes, genetics of species interactions.

I confirm that I have read this submission and believe that I have an appropriate level of expertise to confirm that it is of an acceptable scientific standard.

# Reviewer report: Reviewer response for version 1

**DOI:** 10.21956/openreseurope.17281.r35087

**Published:** 2023-11-03

**Authors:** Jennifer K. Rowntree

**Affiliations:** 1 University of Plymouth, Plymouth, England, UK

I thought this was a really interesting, and as the authors state, understudied, topic. I found the introduction to be well written and that it did a good job of setting the scene for the research. One typo I noted was that in paragraph 4, I think the fourth sentence should read ‘Attempts to experimentally sow…’

 I have similar questions to the first reviewer about how the study species were chosen and urge the inclusion of information from the second paragraph of responses in a revised version of the manuscript. I think it is valid that you chose species that could be distinguished from each other on a plate as you describe, but the reader needs to know what motivated the final choice. I also think including the life-strategy categories according to Heinjo During, as you suggest in your responses is a good idea.

 I found the description of the experimental design rather unclear and spent some time trying to work out the number of replicate plates you had used per treatment (three I believe). I think inclusion of the N per treatment and total number of plates in the methods would help to clarify this. I do think there are some potential issues with this nested design, which can be overcome with the analysis that you have done, but this does need some careful explanation. If I have understood correctly, there are really 3 true replicates per treatment, each with 5 pseudo-replicated colonies. This is much clearer from the figure legends but needs to also be obvious in the main text. Related to this, and for clarity, please include some details of the nested model used to analyse the data, the statistical software used to analyse the data and information on how the significance values were detected. From the figures I assume that these are differences from the control (Dunnett’s test), but this is not necessarily the case.

 In light of the design, I also wondered if there was any growth among colonies on a plate as the plates themselves are not that big. This would obviously lead to some uncertainty in the estimates if that were the case.

 When you set up the control colonies, did you add two drops of spore solution as you do for the competition experiments or were these a single drop (and hence half the density of the competition treatments? Please clarify this in the methods.

 In the results, you mention removing the small brown spores of some species that did not germinate from estimates of total spores. At was stage was this? Was it in the spore solutions or on the plates as you were counting? Were these really non germinating spores?

 In Table 2, can you clarify the final column. I wasn’t sure if this was percent germination or absolute number of spores germinated per plate. There is also an unnecessary hyphen in germin-ated.

 In figures 2 and 3, I think it would help to either put the species names at the top of each panel or provide each panel with a letter and then describe the species it relates to in the legend. I know you have the species names in the legends, but it is not that easy to interpret quickly. See also my previous comment about significant differences and what these actually represent.

 In Figure 3, I was a bit confused whether the y axis was number of gametophores per germinated spores in each treatment (implied from the axes titles) or the number of gametophores per germinated spores in the control, as stated in the legend. Please clarify this.

 Please check the column titles for Table 3 as Mean no. of spores is in there twice, and I assume column 3 is a correlation coefficient. Check also for consistency of decimal point and comma usage in the numbers. I think this table might be better placed earlier in the manuscript, after Table 2 as that is where it is first referred to.

 For figure 4 could you add a legend that shows the respective arrow widths and significance values for ease of interpretation?

 One final question I had was about the number of sporophytes that the spores originated from. This is driven by a question I have about how more widely applicable these findings might be, or whether there is potential for these particular responses to interactions to be driven by the genetic background of the spores.

Is the study design appropriate and does the work have academic merit?

Yes

Is the work clearly and accurately presented and does it cite the current literature?

Partly

If applicable, is the statistical analysis and its interpretation appropriate?

Partly

Are all the source data underlying the results available to ensure full reproducibility?

Yes

Are the conclusions drawn adequately supported by the results?

Yes

Are sufficient details of methods and analysis provided to allow replication by others?

Partly

Reviewer Expertise:

Ex situ bryophyte conservation and in vitro culture of bryophytes, genetics of species interactions.

I confirm that I have read this submission and believe that I have an appropriate level of expertise to confirm that it is of an acceptable scientific standard, however I have significant reservations, as outlined above.

# Response

**Published:** 2024-01-03

**Authors:** Nils Cronberg

**Affiliations:** Biology, Lunds Universitet, Lund, Skåne County, Sweden

*Thanks a lot for being willing to review our manuscript! We are glad that you appreciated our manuscript! Your detailed and constructive suggestions will allow us to make substantial improvements to the text.*   I thought this was a really interesting, and as the authors state, understudied, topic. I found the introduction to be well written and that it did a good job of setting the scene for the research. One typo I noted was that in paragraph 4, I think the fourth sentence should read ‘Attempts to experimentally sow…’ To be changed: ‘Attempts to experimentally sow…’ *This error has apparently gone unnoticed. We will change this in the updated version.*

 I have similar questions to the first reviewer about how the study species were chosen and urge the inclusion of information from the second paragraph of responses in a revised version of the manuscript. I think it is valid that you chose species that could be distinguished from each other on a plate as you describe, but the reader needs to know what motivated the final choice. I also think including the life-strategy categories according to Heinjo During, as you suggest in your responses is a good idea. *We will update this, as already suggested in the response to the first referee.*

 I found the description of the experimental design rather unclear and spent some time trying to work out the number of replicate plates you had used per treatment (three I believe). I think inclusion of the N per treatment and total number of plates in the methods would help to clarify this. I do think there are some potential issues with this nested design, which can be overcome with the analysis that you have done, but this does need some careful explanation. If I have understood correctly, there are really 3 true replicates per treatment, each with 5 pseudo-replicated colonies. This is much clearer from the figure legends but needs to also be obvious in the main text. Related to this, and for clarity, please include some details of the nested model used to analyse the data, the statistical software used to analyse the data and information on how the significance values were detected. From the figures I assume that these are differences from the control (Dunnett’s test), but this is not necessarily the case. *Yes, we used a nested design for the experiments and then a nested (hierarchical) ANOVA for the statistical analyses. The reason for using this approach was that we had no space to place all replicates on separate agar plates. The strength of a nested design is that you can evaluate if there is any effect of the levels in the hierarchy (in this case, 3 agar plates vs 5 spots in each agar plate). There were no such effects. We will point out this in the updated version. We used an Excel spreadsheet provided by the referenced book (McDonald 2014), but this should have been expressed more implicitly. All statistical tests were pairwise, so the only options were either no significance or significance (positive or negative, dependent if facilitation or suppression occurred). So, no tests for contrasts were necessary.* *Changed text (Methods): The spore germination rates of individual species were compared between the single-species cultivation condition (controls) and each of the two pairwise cultivation conditions (i.e., simultaneous and 20-day time-lag treatments) separately (* *Figure 2* *,* *Table S1* *–* *Table S6* *) by nested ANOVAs (* *McDonald, 2014* *) using a spreadsheet template fetched from http://www.biostathandbook.com/nestedanova.html. The gametophore budding rates were in the same way compared for each species between the controls and the budding rates in pairwise culture (* *Figure 3* *,* *Table S1* *–* *Table S6* *). All statistical tests were pairwise, so the only options were either no significance or significance (positive or negative, dependent on facilitation or suppression occurred) in comparison between control and treatment for each species.* *Data was normally distributed, and no transformations were needed. In many cases, the difference between the treatment and the control was quite strong without or almost with no germination in the treatments.*

 In light of the design, I also wondered if there was any growth among colonies on a plate as the plates themselves are not that big. This would obviously lead to some uncertainty in the estimates if that were the case. *No, the colonies did not mix into each other during the time the experiment lasted.*

 When you set up the control colonies, did you add two drops of spore solution as you do for the competition experiments or were these a single drop (and hence half the density of the competition treatments? Please clarify this in the methods. *No, we added just a single drop for the control colonies. We focused on adding the same amounts of spores for each species in the control and competition treatments. So, the amounts of spores were always the same. The additional water when applying two drops was considered insignificant, given that the agar was 98% water, and the additional water was rapidly absorbed by the agar. However, we will make this clearer in an updated version of the manuscript.* In the results, you mention removing the small brown spores of some species that did not germinate from estimates of total spores. At was stage was this? Was it in the spore solutions or on the plates as you were counting? Were these really non germinating spores? *We considered these spores necrotic, so removing them from the calculations was logical. We also checked the germination frequency at the end of the experiment. The germination frequency remained the same (except for Atrichum), so we expect this is not an error source*.

 In Table 2, can you clarify the final column. I wasn’t sure if this was percent germination or absolute number of spores germinated per plate. There is also an unnecessary hyphen in germin-ated. *I see that there is a missing (%) in the column for “Spore germination rate at the end of the experiment”. I will also check if we can get the hyphen removed in the updated version.* In figures 2 and 3, I think it would help to either put the species names at the top of each panel or provide each panel with a letter and then describe the species it relates to in the legend. I know you have the species names in the legends, but it is not that easy to interpret quickly. See also my previous comment about significant differences and what these actually represent. *The significances relate to the growth in controls. So, the question is if each species grows better or worse in combination with another species compared to when it grows alone. We will make this clearer in the legend.* In addition to the legends, bars display mean germination and standard deviation for 15 replicates in a nested design (five replicates within each of three agar plates). " The performance of individual species when growing alone (control) versus in combination with another species (sown simultaneously respectively with a 20-day delay) is statistically tested using separate nested ANOVAs.  Statistical significance levels p=0.05, p=0.01, p=0.001 are marked “*”, “**”, “***”, respectively. *"*

 In Figure 3, I was a bit confused whether the y axis was number of gametophores per germinated spores in each treatment (implied from the axes titles) or the number of gametophores per germinated spores in the control, as stated in the legend. Please clarify this. *The first bar is the control. The other bars reflect the growth under pairwise conditions. Just like for Figure 2, this needs to be better explained.*

 Please check the column titles for Table 3 as Mean no. of spores is in there twice, and I assume column 3 is a correlation coefficient. Check also for consistency of decimal point and comma usage in the numbers. I think this table might be better placed earlier in the manuscript, after Table 2 as that is where it is first referred to. *Yes, you are right. This should be Correlation coefficient. We will update the Table numbering.*

 For figure 4 could you add a legend that shows the respective arrow widths and significance values for ease of interpretation? *Ok. We will consider this. In such a case, it would also be good to explain the colours of the arrows in the figure.*

 One final question I had was about the number of sporophytes that the spores originated from. This is driven by a question I have about how more widely applicable these findings might be, or whether there is potential for these particular responses to interactions to be driven by the genetic background of the spores. *Yes, the spores came from a single sporophyte for each species. We had a passus about this in an earlier version of the ms, but it was removed when we were forced to reduce the number of words to follow a format restriction. This is, of course, a shortcoming of the experiment, but within-species variation would be possible to investigate in the following studies.* *We included a new sentence at the end of the discussion: "* *As our study had a narrow genetic basis, including spores from a single sporophyte for each species, there is also a need to test for variation within species."*

# Reviewer report: Reviewer response for version 1

**DOI:** 10.21956/openreseurope.17281.r35089

**Published:** 2023-10-06

**Authors:** Maxine Watson

**Affiliations:** 1 Indiana University Bloomington, Bloomington, Indiana, USA

This is an interesting, well-written paper, that address – as the authors note – a rarely considered aspect of moss biology. The paper “ Interspecific competition affects spore germination and gametophore development of mosses” examines the early interactions of six species of moss in axenic culture to infer the role of competition in their abilities to establish and initiate populations. 

 My main concern is the study’s small size and thus limited replication, and that their findings of significant results may be owed in part to the difficulty some of the species had in establishing in culture.  None-the-less, their findings are a unique contribution that may well spark others to pursue similar investigations.

 I have another issue of concern and that is their discussion of the role of allelopathy in affecting the relative behaviors of their subject species. Allelopathy as a term is defined differently by different people, and thus is a matter of opinion not yet settled.  However, I think that some of the phenomena that the authors report may be due to the leaching of growth hormones into the medium, and that these hormones may be produced at different times and at different stages of development in the different species.  So I see their effect as an interplay of developmental phenology, time, and proximity.  I realize these are my interests and maybe not those of the authors. At any rate, they may want to take a look at:

 Watson, M.A.  1981.  Chemically mediated interactions among juvenile mosses as possible determinants of their community structure.  J. Chem. Ecol. 7:367-376

 and the references to Bopp’s work therein.

Is the study design appropriate and does the work have academic merit?

Partly

Is the work clearly and accurately presented and does it cite the current literature?

Yes

If applicable, is the statistical analysis and its interpretation appropriate?

I cannot comment. A qualified statistician is required.

Are all the source data underlying the results available to ensure full reproducibility?

Yes

Are the conclusions drawn adequately supported by the results?

Partly

Are sufficient details of methods and analysis provided to allow replication by others?

Yes

Reviewer Expertise:

Plant developmental ecology

I confirm that I have read this submission and believe that I have an appropriate level of expertise to confirm that it is of an acceptable scientific standard.

## References

[1] : Chemically mediated interactions among juvenile mosses as possible determinants of their community structure. *J Chem Ecol* .1981;7(2) : 10.1007/BF00995759 367-76 10.1007/BF00995759 24420482

# Response

**Published:** 2024-01-03

**Authors:** Nils Cronberg

**Affiliations:** Biology, Lunds Universitet, Lund, Skåne County, Sweden

*Thanks a lot for being willing to review our manuscript! We are glad that you appreciated our manuscript!*   This is an interesting, well-written paper, that address – as the authors note – a rarely considered aspect of moss biology. The paper “ Interspecific competition affects spore germination and gametophore development of mosses” examines the early interactions of six species of moss in axenic culture to infer the role of competition in their abilities to establish and initiate populations. 

 My main concern is the study’s small size and thus limited replication, and that their findings of significant results may be owed in part to the difficulty some of the species had in establishing in culture.  None-the-less, their findings are a unique contribution that may well spark others to pursue similar investigations. *Regarding the size of the study, we must put forward that a large amount of work was put into the experiments. With 6 species in pairwise comparison, it means 30 contrasts x 3 petri dishes x 5 spots = 450 spore counts for simultaneous spore germination and equally many for delayed germination. The same number of counts for gametophore emergence. On top of that, 6 x 15 spore counts for controls were repeated twice.*

 I have another issue of concern and that is their discussion of the role of allelopathy in affecting the relative behaviors of their subject species. Allelopathy as a term is defined differently by different people, and thus is a matter of opinion not yet settled.  However, I think that some of the phenomena that the authors report may be due to the leaching of growth hormones into the medium, and that these hormones may be produced at different times and at different stages of development in the different species.  So, I see their effect as an interplay of developmental phenology, time, and proximity.  I realize these are my interests and maybe not those of the authors. At any rate, they may want to take a look at: Watson, M.A.  1981.  Chemically mediated interactions among juvenile mosses as possible determinants of their community structure.  J. Chem. Ecol. 7:367-376 and the references to Bopp’s work therein. *We agree that allelopathy can be defined in different ways. Our results point to the effects of substances excreted by the bryophytes on the ambient environment. It can be discussed whether these substances are just leaked waste products or if they are actively exudated to exclude or promote other species as a means of competition or facilitation. It is clear that developmental phenology could play a role here. In this case, our intention was to study the very first stages of bryophyte development during spore germination and gametophore budding. We will include references to Watson and Bopp in an updated version of the article. In fact, these references were included in an earlier version of the manuscript but were excluded in favour of a broader review by Janice Glime, when we had to reduce the text to meet specific word count restrictions.*

# Reviewer report: Reviewer response for version 1

**DOI:** 10.21956/openreseurope.17281.r35088

**Published:** 2023-10-04

**Authors:** Linda Fuselier

**Affiliations:** 1 University of Louisville, Louisville, Kentucky, USA

Attention to competition in colonization processes related to community composition and the study of interspecific interactions in bryophytes (outside of *Sphagnum*) is lacking in the literature.  As such, this paper presents a needed study that address a gap in our understanding of bryophyte ecology.  That said, studies of competition are not easy to design and implement.  I recommend several changes to the manuscript and encourage authors to consider a more “methods” focused paper.  Ultimately, focusing on the advantages and disadvantages of the methods and species used will be important to subsequent studies.

 Stochastic processes are likely important for bryophyte assemblages that depend on long distance dispersal for colonizers. The first paragraph of the introduction applies mainly to vascular plants rather than bryophytes (see Monteiro et al. 2023 Journal of Ecology).

 Need to define colonization – includes germination and establishment; also some of the “colonizers” that are first to appear on bare soil are not those with small spores but rather large spores – you might want to use During’s life cycle classifications when specifically explaining bryophyte colonization; the life span may be significant to ultimate community composition.

 It is unclear why you chose the species you chose. *Bryum, Ceratodon, Funaria* are early colonizers of bare substrate but *Atrichum, Hypnum* not as much. Please add an explanation of your species choice that supports the comparisons and methods.  Were there any species that you attempted to germinate but did not germinate on the agar and thus decided not to include in the study?  If you wish to connect this to community assemblage composition, it would make sense to use species that are likely to be germinating and establishing together.

 In thinking about a broad readership, you might also explain why you checked for density dependence at the germination stage. Table 1 is nice but if it had to be cut down, the specific sampling coordinates could be omitted; the addition of spore size would be helpful.  I suggest moving spore size to table 1 since information in Table 2 is data collected during the study but spore size is more a characteristic of the species.  I’m assuming that you did not measure the spore size because it is not mentioned in the methods.  Table 3 has a duplicate column heading (no. of spores).

 Germination does not mean establishment.  Although gametophores are produced, depending on the species, the plants may mature earlier or later and produce spores or not and influence what can colonize the area. This will also be influenced by time to germination which was much slower in two of the species you used, both of which were perennials. There are many variables to disentangle so it is difficult to draw conclusions from these particular tests (especially for Atrichum and Hypnum). Results using gametophores are more interesting and very different from those of simply germination; this should be the focus of the discussion.

 The germination rates for some species seem very low even in control situations and for known colonizers. This could indicate a mismatch between agar conditions and naturally occurring germination conditions.  

 The conclusions accurately point out that this is a study paving the way for additional studies of exudation, facilitation and competition at this stage of assembly. However, any claims about rhizosphere exudation are a stretch – this was not specifically tested here.

Is the study design appropriate and does the work have academic merit?

Partly

Is the work clearly and accurately presented and does it cite the current literature?

Yes

If applicable, is the statistical analysis and its interpretation appropriate?

I cannot comment. A qualified statistician is required.

Are all the source data underlying the results available to ensure full reproducibility?

Partly

Are the conclusions drawn adequately supported by the results?

Partly

Are sufficient details of methods and analysis provided to allow replication by others?

Partly

Reviewer Expertise:

Bryophyte ecology

I confirm that I have read this submission and believe that I have an appropriate level of expertise to confirm that it is of an acceptable scientific standard, however I have significant reservations, as outlined above.

# Response

**Published:** 2024-01-03

**Authors:** Nils Cronberg

**Affiliations:** Biology, Lunds Universitet, Lund, Skåne County, Sweden

Thanks for insightful and constructive suggestions! Attention to competition in colonization processes related to community composition and the study of interspecific interactions in bryophytes (outside of *Sphagnum*) is lacking in the literature.  As such, this paper presents a needed study that address a gap in our understanding of bryophyte ecology.  That said, studies of competition are not easy to design and implement.  I recommend several changes to the manuscript and encourage authors to consider a more “methods” focused paper.  Ultimately, focusing on the advantages and disadvantages of the methods and species used will be important to subsequent studies. *We already have a rather strong focus on methodology, and we stress in the concluding remarks that we pave the way for novel experimental designs based on our study. We also briefly mention the limitations of our approach. We have expanded the part of the discussion where we discuss the limitations in a new version of the article.*

 Stochastic processes are likely important for bryophyte assemblages that depend on long distance dispersal for colonizers. The first paragraph of the introduction applies mainly to vascular plants rather than bryophytes (see Monteiro et al. 2023 Journal of Ecology). *Yes, the introduction is intended to broadly introduce the research area for plants and then focus on bryophytes. The Monteiro publication was unavailable when we finished our ms, but we will incorporate it into an updated text version. As a matter of fact, a major conclusion from the study of Monteiro et al is that* *at fine scale, environmental filtering prevails in stressful habitats, while competitive interactions appear more important in more favourable conditions. We have also found another quite important reference on this topic, which will be incorporated into the updated version: Barbé M, Fenton N J, Bergeron Y: So close and yet so far away: long-distance dispersal events govern bryophyte metacommunity reassembly. J Ecol. 2016;104(6):1707-1719. https://doi.org/10.1111/1365-2745.12637*

 Need to define colonization – includes germination and establishment; also some of the “colonizers” that are first to appear on bare soil are not those with small spores but rather large spores – you might want to use During’s life cycle classifications when specifically explaining bryophyte colonization; the life span may be significant to ultimate community composition. *All the included species frequently have sporophytes and small spores, so they are likely to be present in a common spore cloud during early autumn. It is possible that species with large spores and shorter dispersal capacity (shuttle species, sensu During) are more competitive. We did not include any of those species, which would be interesting to study in future experiments. We have added a reference to this in the final part of the discussion.*

 It is unclear why you chose the species you chose. *Bryum, Ceratodon, Funaria* are early colonizers of bare substrate but *Atrichum, Hypnum* not as much. Please add an explanation of your species choice that supports the comparisons and methods.  Were there any species that you attempted to germinate but did not germinate on the agar and thus decided not to include in the study?  If you wish to connect this to community assemblage composition, it would make sense to use species that are likely to be germinating and establishing together. *Yes, we are aware that the included species differ in various respects, such as life span duration and life strategies. We chose to include species representing different taxonomic groups and different life strategies because this seemed to be a logic first step for a pioneering study. Also, we had to use species that had spores and gametophore buds, which could be pairwise discriminated (by size, shape and colour) on agar plates in a microscope.* *We did not test any other species.* *As stated under “Study species”, the species included in the study do not occur in any specified community, although they can sometimes be found growing adjacent to each other in southern Sweden. All are growing on the ground (Hypnum also on other substrates) and at similar pH levels.* *At this stage, there is no data to assume that species forming communities have stronger or weaker pairwise interactions. Likewise, it is not evident how species representing different life strategies would differ in terms of interactions during germination. We have modified the text under the concluding remarks to make this clearer.:* *"We observe considerable differentiation in interactions between species, but the data set is too limited to draw any conclusions related to phylogenetic lineages, habitats or life forms. We avoided focusing on any particular moss community because naturally co-occurring species could have evolved specific adaptations – our results should be seen as examples of more generalized responses."* *The most important point is that the included species all produce abundant sporophytes and release their spores during the same period in the late summer. This is pointed out under “Study species” and will be further emphasized in the revised version. The earlier mentioned study by Barbé et al. supports our conception that abundant spore production is important for the regional spore cloud and the chance of encounter at spore deposition.*

 In thinking about a broad readership, you might also explain why you checked for density dependence at the germination stage. *We have made this clearer by a small addition:* *"The exact number of spores in inoculates was somewhat variable. To check if this could be a source of error, we tested for density-dependent spore germination in the control treatment…"* Table 1 is nice but if it had to be cut down, the specific sampling coordinates could be omitted; the addition of spore size would be helpful.  I suggest moving spore size to table 1 since information in Table 2 is data collected during the study but spore size is more a characteristic of the species.  I’m assuming that you did not measure the spore size because it is not mentioned in the methods.  Table 3 has a duplicate column heading (no. of spores). *We avoid making unnecessary changes in the tables because of the risk of introducing new errors. However, the duplicate column (no. of spores) is a mistake, the second column should be correlation coefficient.*

 Germination does not mean establishment.  Although gametophores are produced, depending on the species, the plants may mature earlier or later and produce spores or not and influence what can colonize the area. This will also be influenced by time to germination which was much slower in two of the species you used, both of which were perennials. There are many variables to disentangle so it is difficult to draw conclusions from these particular tests (especially for Atrichum and Hypnum). Results using gametophores are more interesting and very different from those of simply germination; this should be the focus of the discussion. *We disagree to some extent here. Germination is the first step of colonization after dispersal. The spore germination tests were performed both with simultaneous sawing and with a delay. The results are similar but stronger with a delay. We are pointing out the limitations in the interpretation of Atrichum.*

 The germination rates for some species seem very low even in control situations and for known colonizers. This could indicate a mismatch between agar conditions and naturally occurring germination conditions. *Yes, maybe also the fact that we stored spores in a fridge in a water solution could be responsible for the germination rates. However, we tested the germination frequency at the start and end of the experiments and it differed only for Atrichum, so it should not affect the results for the other species.*

 The conclusions accurately point out that this is a study paving the way for additional studies of exudation, facilitation and competition at this stage of assembly. However, any claims about rhizosphere exudation are a stretch – this was not specifically tested here. Ok. We reformulate a bit. “ We envision that bryophyte exudation might compare to rhizosphere exudation…”. We actually have some data on the chemical composition of exudates from bryophytes, which is quite diverse. Also, other recent publications point in this direction, but remember where you read it first!

# Reviewer report: Reviewer response for version 1

**DOI:** 10.21956/openreseurope.17281.r34743

**Published:** 2023-08-31

**Authors:** Marko S. Sabovljevic

**Affiliations:** 1 Institute of Botany and Botanical Garden Jevremovac, Faculty of Biology, University of Belgrade, Belgrade, Serbia

The study itself is pioneer and thus very interesting. However, the design remains blurred and thus the results seems to be rather unsupported. There are too many assumptions. Some of the species are ecologically unrelated and not in natural competition at all. Some have naturally rapid and short life span and the others are perennial, thus completely different life strategies suggesting quick vs. slow germination rate cannot advance in nature always. Some literature can be found on these phenomena.

 Also, it is unclear how did the authors distinguished the spores and protonema of different species once you mix them. Are you sure the one protonema is originated from one spore when calculating bud formation. What about tmema cells, ruptures or even brachycites? Not mentioned at all. When did caulonema occur?

 Exogenously substances in different concentrations can affect some process positively and other negatively. These can be the answer for many of your assumptions as shown already by Martin Bopp in the 1980s. Also, the effect of spores, protonema or bud stages is not linked and easy to followed in the manuscript. 

 Life strategies, life forms and finally biological features of various mosses tested here including life span are no considered.

 What about photoblastic, light quality and/or recalcitrance in spore germination? There are some of these phenomena mentioned in the previous literature not mentioned in this study.

 Also, spore germinability is not equal to spore viability. Some spores are prepared to remain in natural spore banks and to be transferred by various vectors in nature! Not considered here.

 Author mentioned that they are aware of understudied bryophyte spore biology but it is not considered within the manuscript. Thus it makes the good idea rather preliminary.

 Thus, I suggest major revision and full rewrite of the manuscript.

Is the study design appropriate and does the work have academic merit?

Partly

Is the work clearly and accurately presented and does it cite the current literature?

No

If applicable, is the statistical analysis and its interpretation appropriate?

I cannot comment. A qualified statistician is required.

Are all the source data underlying the results available to ensure full reproducibility?

Partly

Are the conclusions drawn adequately supported by the results?

Partly

Are sufficient details of methods and analysis provided to allow replication by others?

No

Reviewer Expertise:

Bryophyte Biology

I confirm that I have read this submission and believe that I have an appropriate level of expertise to confirm that it is of an acceptable scientific standard, however I have significant reservations, as outlined above.

# Response

**Published:** 2023-09-08

**Authors:** Nils Cronberg

**Affiliations:** Biology, Lunds Universitet, Lund, Skåne County, Sweden

**Author response to Reviewer 1 Marko S. Sabovljevic**

**Thanks a lot for being willing to review our manuscript! We appreciate that you checked our manuscript swiftly with many thoughtful suggestions! The referee texts are written in italic text and our responses in bold text. Changed texts are underlined. We will make a revised version of the manuscript based on the suggestions communicated here. We will do this when we have received reports from additional referee(s).**

*The study itself is pioneer and thus very interesting. However, the design remains blurred and thus the results seem to be rather unsupported. There are too many assumptions. Some of the species are ecologically unrelated and not in natural competition at all. Some have naturally rapid and short life span and the others are perennial, thus completely different life strategies suggesting quick vs. slow germination rate cannot advance in nature always. Some literature can be found on these phenomena.*

**Yes, we are aware that the included species differ in various respects such as life span duration and life strategies. We chose to include species representing different taxonomic groups and different life-strategies because this seemed to be a logic first step for a pioneering study. Also, we had to use species that had spores and gametophore buds which could be pairwise discriminated (by size, shape and colour) on agar plates in a microscope. As stated under “Study species” the species included in the study do not occur in any specified community, although they can sometimes be found growing adjacent to each other in southern Sweden. All are growing on the ground (Hypnum also on other substrates) and at similar pH levels. At this stage, there is no data to assume that species forming communities have stronger or weaker pairwise interactions. Likewise, it is not evident how species representing different life strategies would differ in terms of interactions during germination. The most important point is that the included species all produce abundant sporophytes and release their spores during the same period in the late summer. This is pointed out under “Study species” but needs to be further emphasized in a revised version. The spores that become airborne are likely to mix with each other in the air, much like wind-dispersed pollen. During rainfall, they will be washed to the ground and are likely to encounter on potential colonization spots on bare ground.**

*Also, it is unclear how did the authors distinguished the spores and protonema of different species once you mix them. Are you sure the one protonema is originated from one spore when calculating bud formation. What about tmema cells, ruptures or even brachycites? Not mentioned at all. When did caulonema occur?*

**As explained above, the spores could be discriminated by differences in size, shape and colour on agar plates in a microscope. We used two variables in the analyses: spore germination frequency and the frequency of gametophore buds relative to the number of spores germinated. We scored the spore germination and gametophore numbers at fixed time points. It was difficult, but doable, to identify the spores in the species pairs. It was somewhat easier to identify the gametophore buds. We included pictures of germinating spores and gametophore buds for all species in an earlier version but decided to exemplify with just one species in the present version. We did not score any details of the stages between germination and gametophore bud formation. This could have been interesting but would have required an enormous amount of work, given the number of replicates we had in the study. For this reason, we did not try to compare the development of protonemata between species, nor did we quantify the amounts of protonemata or set a timepoint when the switch between chloronemata and caulonemata took place. This would be interesting to investigate in a subsequent study. It is clear that one spore can produce more than one gametophore bud, but it was not our focus to investigate the number of gametophores produced by individual spores. This would have required us to work with single-spore cultures. We scored the number of gametophores in each replicate spot and divided this number by the number of germinated spores for the same species (see Table 2 for single-spore tests and Table S1-S6 for pairwise tests). In most cases, the mean number of gametophores per germinated spore was less than one. The number of emerging gametophores will likely increase over time, but we did not check that. We did however check for density dependence.**

*Exogenously substances in different concentrations can affect some process positively and other negatively. These can be the answer for many of your assumptions as shown already by Martin Bopp in the 1980s. Also, the effect of spores, protonema or bud stages is not linked and easy to followed in the manuscript.*

**We are aware of the studies by Martin Bopp testing the effect on protonema by different plant hormones. We have included some more recent references on this topic to Vesty et al. 2016, Decker et al. 2006 and Reski 1998. The topic is also reviewed in the referenced texts by Glime. However, we will include references to the research of Bopp in a new version of the manuscript and consider how the research could aid in the interpretation of our data. However, as far as we know he has not studied interspecies interaction between protonemata.**

**A reference to Maxine Watsons work is also relevant in the introduction (changes underlined):**

 Plant allelopathy is defined as interference concerning growth due to chemical interactions between plants and other organisms, mediated by the release of plant-produced bioactive or toxic specialized metabolites referred to as allelochemicals ( Latif *et al.*, 2017). Such metabolites could have beneficial (stimulatory) or detrimental (inhibitory) effects on target organisms ( Cheng & Cheng, 2015; Zhang *et al.*, 2021). Interactions between moss species in their earliest growth stages have received little attention (Watson, 1981) . Based on studies by (Bopp, 1959) and own observations Watson (1981) suggested that “differences among individuals (taxa) in arrival time and susceptibility to leaked chemicals would appear likely to effect the outcome of competitive interactions among them”.  As for the discussion: Negative density-dependent effects occur commonly in vascular plants when crowded seedlings compete for resources and it seems that the stronger this mechanism is, the higher the plant diversity becomes at the global scale ( LaManna *et al.*, 2017). Negative density-dependent effects are likely to occur in the early colonization of bryophytes. For example, it is known that high spore densities may cause the failure of caulonema differentiation (self-inhibition) ( Zamfir & Goldberg, 2000), which might prevent intraspecific competition among protonemata. Also, Bopp (1959) and later Watson (1981) found that single spores of *Funaria hygrometrica* sown at some distance formed separate protomema that kept apart at encounter, whereas spores sown together freely merged to form a common protonema. We did not see density-dependency in our study at the applied densities, except for *A. undulatum*, which displayed negative density-dependence for spore germination and positive density-dependence at gametophore budding ( Table 3), so this factor is unlikely to affect the results. Bopp, M. (1959). Versuche zur analyse von Wachstum und Differenzierung des Laubmoosprotonemas. *Planta, 53*, 178-197. Watson, M. A. (1981). Chemically mediated interactions among juvenile mosses as possible determinants of their community structure. *Journal of Chemical Ecology, 7*(2), 367-376.

*Life strategies, life forms and finally biological features of various mosses tested here including life span are no considered.*

*What about photoblastic, light quality and/or recalcitrance in spore germination? There are some of these phenomena mentioned in the previous literature not mentioned in this study.*

**The growth forms and life strategies are briefly accounted for in Table 1. We will expand on this and more strictly follow the life-strategy categories according to Heinjo During. This will also be followed up more in depth in the discussion of an updated version of the manuscript. However, it is difficult, based on the limited number of species in the data set, to trace patterns based on the life-strategy concept. The study was performed in a growth chamber. With respect to light, we used a light emitting plasma (LEP) fixture which has a light spectrum which is similar to sun light. The light intensity was fairly low, but uniform, at level of the table we used for cultures. We were aware of the risk for position effects, so the Petri dishes were randomly redistributed at regular intervals during the study. This is described under “Experimental design and data analysis”. The important point was that all replicates were subject to the same conditions to avoid interfering effects of phenomena mentioned by the reviewer, such as photoblastic differences, differential response to light quality or dormancy which surely occur among the species. In the statistical treatment we did not see any systematic effects relating to the Petri dish level or the individual spot within Petri dish.**

*Also, spore germinability is not equal to spore viability. Some spores are prepared to remain in natural spore banks and to be transferred by various vectors in nature! Not considered here.*

**Yes, good point. As the spore germination was far from complete in most of our species, dormancy is one possible explanation. We should stress this. We cite a study by Vesty et al 2016 which states (partially based on other studies) that primary dormancy lacking in several bryophytes. We will look more into this, but as stated by Glime, there are few studies of dormancy in bryophytes and even fewer that test the potential for differential degree of dormancy in individual sporophytes. On the other hand, there are many studies indicating diaspore banks in the soil, sometimes persistent during decennia, but this is somewhat outside the scope of this study. However, primary dormancy is unlikely to affect our results. We tested the germination frequency of all species in single-species agar plates at the start as well as the end of the experiment. We did this to check for changes in spore germinability over time. The spore germination rate did not differ, except for Atrichum which dropped considerably in spore germination frequency, probably due to spore necrosis during storage.**

**As for the discussion:**

 Except for *A. undulatum*, the germination rate of spores from other species did not decrease after two months storage in 4°C in darkness. The spore germination rate was relatively low for the studied species (except *L. pyriforme*) in single-species cultures, maybe due to dormancy induced by storage, but this is unlikely to affect the results since the germination rate remained stable over time (except for *A. undulatum*). Data on the longevity of moss spores seems scarce ( Pence, 2004) and most studies have tested survival in dry conditions ( Proctor *et al.*, 2007), so it is an open question how long they remain viable under cool and dark conditions and to what extent it differs within species.

 Author mentioned that they are aware of understudied bryophyte spore biology but it is not considered within the manuscript. Thus it makes the good idea rather preliminary.

**Yes, bryophyte spore biology is a generally neglected research area. In our introduction and discussion, we concentrate on interactions between bryophyte species at germination as this is the focus of our study.**
